# Clinical, Pathological and Molecular Characteristics of Chilean Patients with Early-, Intermediate- and Late-Onset Colorectal Cancer

**DOI:** 10.3390/cells10030631

**Published:** 2021-03-12

**Authors:** Karin Alvarez, Alessandra Cassana, Marjorie De La Fuente, Tamara Canales, Mario Abedrapo, Francisco López-Köstner

**Affiliations:** 1 Oncology Center, Clinica Universidad de Los Andes, Santiago 7620157, Chile; kalvarez@clinicauandes.cl; 2 Joint Doctoral Degree Program in Medical Sciences, Faculty of Medicine, Universidad de Chile, Santiago 8380453, Chile; ccassana@clinicalascondes.cl; 3 Coloproctology Unit, Clinica Las Condes, Santiago 7591047, Chile; mabedrapo@clinicalascondes.cl; 4 Academic Direction, Clinica Las Condes, Santiago 7591047, Chile; mkdelafu@gmail.com; 5 Cancer Institute, Clinica Las Condes, Santiago 7591047, Chile; tcanales@clinicalascondes.cl; 6 Faculty of Medicine, Universidad de Chile, Santiago 8320000, Chile

**Keywords:** colorectal cancer, age of onset, cancer diagnosis, germ-line mutations

## Abstract

Colorectal cancer (CRC) is the second most frequent neoplasm in Chile and its mortality rate is rising in all ages. However, studies characterizing CRC according to the age of onset are still lacking. This study aimed to identify clinical, pathological, and molecular features of CRC in Chilean patients according to the age of diagnosis: early- (≤50 years; EOCRC), intermediate- (51–69 years; IOCRC), and late-onset (≥70 years; LOCRC). The study included 426 CRC patients from Clinica Las Condes, between 2007 and 2019. A chi-square test was applied to explore associations between age of onset and clinicopathological characteristics. Body Mass Index (BMI) differences according to age of diagnosis was evaluated through t-test. Overall (OS) and cancer-specific survival (CSS) were estimated by the Kaplan–Meier method. We found significant differences between the age of onset, and gender, BMI, family history of cancer, TNM Classification of Malignant Tumors stage, OS, and CSS. EOCRC category was characterized by a family history of cancer, left-sided tumors with a more advanced stage of the disease but better survival at 10 years, and lower microsatellite instability (MSI), with predominant germline mutations. IOCRC has shown clinical similarities with the EOCRC and molecular similarities to the LOCRC, which agrees with other reports.

## 1. Introduction

Colorectal cancer (CRC) is the third most common neoplasm and the second leading cause of death in developed countries [1]. In Chile, DEIS-MINSAL (Department of Statistics and Health Information, Ministry of Health, 2018) [2] observed an increase of 54% of deaths by CRC (from 3554 in 2009 to 5472 in 2018), being the third most common cause of cancer mortality (manuscript in preparation). A previous study analyzing data between 1983 and 2008 demonstrated a progressive increase in mortality rates (116%) [3], probably due to risk factors related to increases in life expectancy, urban residence, lifestyle, nutrition, and ethnic background, constituting a relevant public health issue.

The vast majority of CRC cases (80%) are classified as sporadic [4,5]. The remaining cases (10–20%) have a family history [6,7], approximately 10% of which are derived from a moderately penetrating hereditary susceptibility and possibly interacting with environmental factors [8]. In approximately 5–10% of cases, the disease is caused by a highly penetrant inherited syndrome [6,9], the most common being Lynch Syndrome (LS), which accounts for 3–5% of CRC cases [10,11,12].

Age at diagnosis is one of the main risk factors and is considered a potential predictor of the development of CRC [4,5,6,7,9,10,13,14,15,16,17,18,19,20,21,22]. In the last 20 years, the incidence of sporadic early-onset CRC (EOCRC) has been increasing steadily, with advanced stages of the disease at the diagnosis [7,9,15,16,17,23] and a worse prognosis and survival rate [10,12,24]. Around 10–11% of colon cancer and 18% of rectal cancer occur in patients under 50 years, respectively [15]. Furthermore, 7% of cases are diagnosed before the age of 40 [15]. Likewise, an exponential increase in the incidence of colon and rectal cancer in people under 35 years is predicted by 2030, as much as 90% and >140%, respectively [8,16], with an increase in tumors of the rectosigmoid junction [14,17].

There is evidence that genetic, biological, and pathological characteristics differ according to cancer onset and location of the tumor as well [4,6,8,9,23], which implies different carcinogenic pathways and a more aggressive phenotype [20]. From a molecular point of view, EOCRC presents different molecular features in comparison to late-onset colorectal cancer (LOCRC) [6,16], with the identification of different oncogenic signaling pathways (microsatellite and chromosomal instability, CpG island methylator phenotype), as well as punctual markers such as *KRAS* mutation up to 54% and alterations in specific chromosomal regions [6,13,14,16,23,25,26]. From the pathological point of view, a higher frequency of tumor in the left colon is currently described, followed by the rectum, with poor histological differentiation and a greater mucinous component and signet-ring cells [4,6,9,14,17,19,20,23,26].

Several efforts have been made towards clinicopathological characterization of CRC in Latin America [5,7,27,28], although there remains to be universal access to the genetic testing of all populations from our region or a better clinical characterization, which is relevant for the surveillance and management of the patients. To our knowledge, in Chile there are no comparative characterization studies of sporadic CRC. The present study aimed to identify the clinicopathological and molecular characterization of CRC in Chilean patients according to the age of diagnosis as the main criterion of classification. The identification of differences between early-, intermediate-, and late-onset cancers will be relevant for future decision-making in prevention, screening, and treatment strategies to optimize outcomes in our population.

## 2. Materials and Methods

A retrospective, observational, and analytical study was carried out. Clinical, pathological, and molecular characteristics of patients with diagnosis of CRC who were treated at Clinica Las Condes (Santiago, Chile) between 2007 and 2019 were collected.

### 2.1. Colorectal Cancer Registry from Clinica Las Condes

A total of 426 Chilean patients with CRC were recorded prospectively in our registry, which was started in 2007. The collected information included age at onset, birth date, death date, pathology data, molecular data, the results of germline testing, and personal and family history of cancer. All patients were regularly followed up, including the review of death certificates in the national registry of Chile to record mortality with the specific cause of death.

The study was conducted in accordance with the Declaration of Helsinki, and the protocol was approved by the Ethics Committee of Clinica Las Condes. All patients gave informed consent for their participation in the study.

### 2.2. Study Population

In order to analyze the CRC patients, we defined three groups:

- Early-onset colorectal cancer (EOCRC): ≤50 years
- Intermediate-onset colorectal cancer (IOCRC): 51–69 years
- Late-onset colorectal cancer (LOCRC): ≥70 years

The clinical characteristics studied were age, gender, type of cancer (colon or rectum), and anatomic location (right colon, left colon, or rectum), BMI, TNM (Tumor Nodes Metastasis—Classification of Malignant Tumors stage), and mortality. Moreover, personal history of other synchronous or metachronous malignant tumors; a history of first-degree relatives’ cancer such as CRC, cancers related to LS—ovary, uterus, gastric, kidney, small intestine, skin—and cancers not related to LS—liver, lung, and others—and LS criteria (Amsterdam, Bethesda, Universal Tumor Screening) fulfillment. With respect to molecular characteristics, we searched for tumor markers—MSI grade, BRAF mutation, *KRAS* mutation, expression of repair proteins (MLH1, MSH2, MSH6, and PMS2), and germline mutations.

### 2.3. Statistical Analysis

The clinical characteristics were described using frequency distributions for categorical variables. A chi-square test was applied to explore associations between the age of onset and the clinical variables. All reported *p*-values were two-sided, and statistical significance was set to the 95% level (*p* < 0.05).

Overall (OS) and cancer-specific survival (CSS) were estimated by the Kaplan–Meier method. The results were obtained with a confidence interval of 95% and a *p*-value < 0.05 was considered statistically significant. The analyses were made using R Project version 3.5.3.

Regarding ethical approval and consent to participate, all genetic tests were done with appropriate informed consent according to local requirements for healthcare and/or research.

## 3. Results

Between 2007 and 2019, a total of 426 patients with CRC were registered. Comparative results between early-, intermediate- and late-onset CRC categories are shown in Table 1.

### 3.1. Clinical, Pathological, and Familial Features

From 426 patients registered, 72 (17%) belonged to the EOCRC group, 214 (50%) to IOCRC, and 140 (33%) to LOCRC. The average age at CRC diagnosis was 43 years (range 29–50), 61 years (range 51–69) and 79.8 years (range 70–98), respectively. We found a significant association between the age of onset and gender (*p* = 0.0031) in which the IOCRC group exhibited a major frequency of males (63%) (Table 1). The location of the tumor showed significant differences according to the age of onset, with a predominance of left-sided tumors in the younger groups (56.9% in EOCRC and 51.9% in IOCRC, respectively) compared to the older group who mainly developed right-sided tumors (43%) (*p* = 0.0132). Furthermore, EOCRC patients presented more advanced disease at the diagnosis in comparison to intermediate- and late-onset CRC (stage IV, 28%, 17%, and 12%, respectively; *p* = 0.0174). Additionally, differences in BMI were identified according to the age of diagnosis (*p* = 0.0009), identifying a significant increase in the BMI of IOCRC (26.35) vs. EOCRC (24.4) (Dunn’s Multiple Comparison Test; *p* < 0.05) (Table 1).

**Figure 1:**
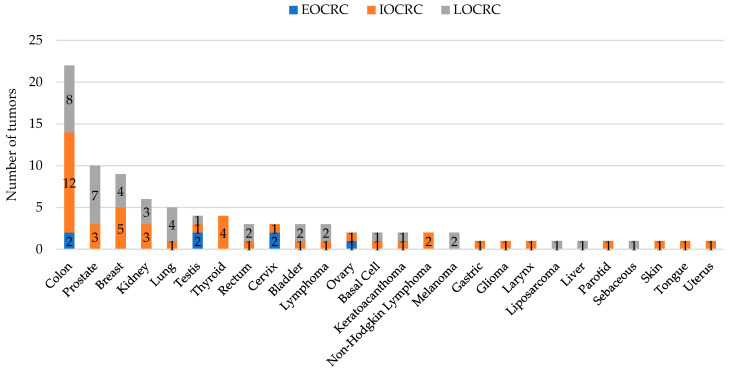
Personal history of other synchronous or metachronous malignant tumors: Tumor type frequency according to the age of onset.

The personal history of CRC and other tumors related or not related to LS (synchronous or metachronous) increased according to age (Table 1). We observed an increase of cases with two or more tumors from 9.7% in EOCRC to 18.7% in IOCRC and 21.4% in LOCRC. The spectrum and frequency of tumors are shown in Figure 1. There was no significant association between the age of onset and the presence of multiple tumors (*p* = 0.5667). Concerning the family history of cancers, we did not find significant differences when analyzing the age of onset and the subcategories of CRC, Lynch-related, or other cancers. However, when we grouped the total family history of cancer, there was a significant association with predominance in the younger categories (22.2% in EOCRC, 29.4% in IOCRC) compared to 17.1% in LOCRC (*p* = 0.0256) (Table 1).

During this period of study, the distribution of CRC cases showed a stable trend according to the age of onset over the years (Table 2).

### 3.2. Molecular Analysis

**Figure 2:**
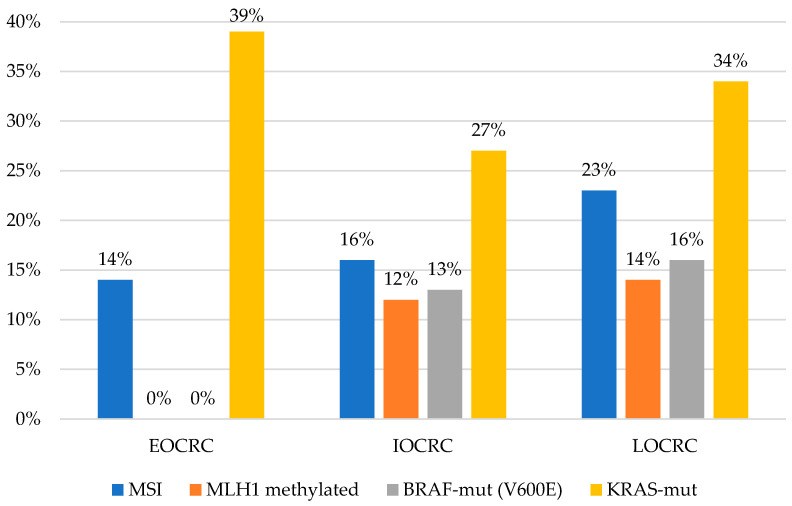
Distribution of molecular features of tumors according to the age of onset.

A total of 74 of the 416 (17.7%) tested tumors showed MSI, whose highest prevalence was observed in LOCRC (31/137; 23%) followed by IOCRC (33/210; 16%) and EOCRC (10/69; 14%) (*p* = 0.1897). In accordance, a predominance of methylated MLH1 was seen in the LOCRC (14%; 6/42) and IOCRC (12%; 8/69) compared to EOCRC (0%; 0/12). There was no significant difference between them (*p* = 0.1897 and *p* = 0.3876, respectively) (Table 3 and Figure 2).

According to BRAF mutations (V600E), a gradual increase was seen between categories (0% in EOCRC (0/28), 13% in IOCRC (12/91), and 16% in LOCRC (9/55)); however, no significant differences were found (*p* = 0.1297). Likewise, according to *KRAS* mutations, there were no significant differences between categories (*p* = 0.3225), although we found a greater number of cases in EOCRC (39%; 13/33) and LOCRC (34%; 21/61) compared to IOCRC (27%; 27/101) (Table 3 and Figure 2).

Of the established groups, most patients who met Amsterdam criteria belonged to EOCRC (9.7%; 7/72) versus 2.8% (6/214) in IOCRC and 0.7% (1/140) in LOCRC. Similarly, patients with CRC and colonic polyposis were found only in the EOCRC group (3/72; 4.2%). Germline mutations were predominant in EOCRC (64%; 9/14) followed by IOCRC (27.7%; 5/18), identifying four MLH1 mutations, two MSH2, two EPCAM deletion, three PMS2, and three APC (Table 3).

### 3.3. Survival Analysis

**Figure 3:**
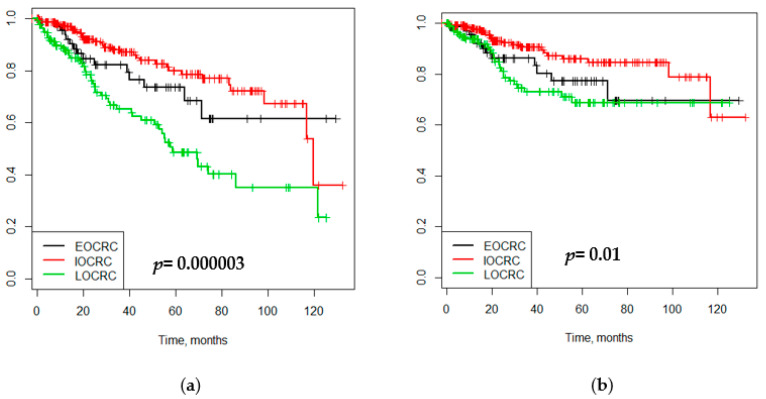
Survival analysis. (**a**) Overall survival by age of onset; (**b**) cancer-specific survival by age of onset.

The OS and CSS curves were performed. According to the age of onset, significant differences in OS and CSS were seen for each group, with *p* = 0.000003 and *p* = 0.01, respectively. (Figure 3).

Five- and ten-year survival analysis showed that EOCRC patients have better survival to ten years or more after CRC diagnosis, with 61.5% in OS (95% CI 45.2–83.7) and 69.4% in CSS (95% CI 53.4–90.3) (Table 4).

## 4. Discussion

The incidence and mortality of CRC in patients under 50 years is increasing steadily and has become a hazard to public health worldwide [15,25,28,29]. In Chile, during the last 20 years, the crude mortality rate has shown a gradual increase, both in the total population and in the group of patients <50 years [3], which is consistent with the other Latin American countries [5,7,28]. In this context, the identification of the clinical, pathological, and molecular features becomes essential to improve the screening, diagnosis, and treatment strategies in the patients [15,23].

Interestingly, during the period of this study, despite finding a higher percentage of EOCRC than the non-Hispanic countries (17% versus 10% approximately) [7,9,15,16,17,23], the distribution of CRC cases showed a stable trend according to the age of onset, which contrasts with the data published worldwide [15,25,28,29].

In the present study, we found significant differences according to the age of onset of CRC. This is consistent with the results described by Bohorquez et al. from Colombia, with a greater incidence of EOCRC (26.5%) in the Latin American population [28]. The possible reasons may be related to changes in diet and sedentary lifestyle in the west over the last decades and highlight the need to take action over strategies to early diagnosis [5].

It should be noted that half of the cases of our registry belonged to the IOCRC group, which defines a group that several studies do not recognize as a subclassification itself but seems to be a transitional one between early- and late-onset CRC [5,23,25,30]. Moreover, the IOCRC group show a higher representation of male gender and higher BMI compared to other groups. On one hand, the higher CRC rate in males has been reported previously [6,9,18] and might be related to a higher vulnerability to environmental threats such as alcoholic beverages, smoking, and poor dietary patterns [8]. On the other hand, it is known that a 5 inch increase in BMI increases CRC risk by 13–18% [10,14,16] and is associated with dysbiosis and inflammation as carcinogenic mechanisms [25]. These findings are consistent with those reported by Arriba et al., who suggest that the IOCRC group bore more resemblance to the EOCRC because both groups share some characteristics such as a predominance of left-sided tumors and a tendency to present more advanced disease proportionally inverse to age [25]. In fact, it is recognized that sporadic tumors in the younger population are more often located in the left colon and rectum and are diagnosed in more advanced stages [4,5,6,7,10,15,16,17,18,19,23,29] which would correspond to different gene expression and frequency of mutations [4,6,8,9,17,19,23,28]. 

It is known that the personal history of other primary tumors, as well as the family history of cancer, is a determining factor of CRC risk, for the identification of hereditary variants and the screening. In this study, we found a greater trend of multiple primary tumors as age increases, without significant differences, which contrasts with the reported by Liang et al., who recognize that EOCRC patients are more likely to present with synchronous (5.8% vs. 1.2%) and metachronous (4.0% vs. 1.6%) tumors compared to older patients [8,20,31]. This may be related to the aging process and the presence of low-penetrance mutations in these patients.

With respect to the family history of cancer, we found significant association according to the age of onset with predominance in the IOCRC followed by EOCRC when we analyzed the total number of cancers, which agrees with the evidence that family history remains one of the strongest determinants of CRC risk, indicating a possible role of a genetic predisposition to moderate or highly penetrating mutations [6,8,9,16,17,18,28,29].

From a molecular point of view, CRC has distinctive molecular features according to the age of onset. In this study, we found a higher frequency of MSI tumors in the LOCRC group, which is supported by previous research where most of the EOCRC exhibit microsatellite-stable phenotypes [16,19,29]. In this LOCRC group, a higher prevalence of MSI tumors located on the right side was found, also concordant with that previously reported [8,19,29,32]. Although there were no statistically significant differences, we found that the MSI identified in LOCRC and IOCRC is related to hypermethylation of MLH1 in the LOCRC, and as expected, the hereditary germline mutations in MMR genes were predominant in EOCRC (64%); findings that are consistent with the literature [8,19]. In fact, there is evidence that the early group with pathogenic germline variants usually present mutations in MMR genes that are associated with LS [14,17,33]. Unfortunately, not all the patients of our registry that met the criteria for genetic analysis were studied for characterization of germline mutations, therefore, we cannot make cross-comparisons between them. In general, Chile and the rest of Latin American countries have different realities, so the access to genetic studies is restricted to those patients who can pay for them and where there are high-risk units with professionals who perform genetic counseling. The implementation of a hereditary cancer registry allows the identification of individuals at risk, genetic testing, genetic-clinical counseling, early detection of cancer, education of families, and training of professionals, thereby reducing the mortality and morbidity of LS patients [34].

Regarding the other genetic markers evaluated—BRAF and *KRAS*—we found a lower prevalence of BRAF V600E mutations in EOCRC, which is consistent with the previously reported when compared to LOCRC [4,8,17,20]. Although we did not find significant differences in the presence of *KRAS* mutations according to the age of onset, we found an increasing trend as age increases, similar to previously reported, with a numerically but not significantly lower prevalence in younger patients [4,25,33,35,36].

Finally, we found that the OS and CSS were better in the IOCRC group at 5-year survival but they are better in the EOCRC group at 10-year survival; and worse in the LOCRC one. Although younger patients are more likely to present with more advanced stages [20], there is the overall consistency of results across multiple study populations supporting better survival in EOCRC patients [6,18,20,25] when compared to LOCRC. Our results are consistent with what was found by Arriba et al. with a similar behavior between the early- and the intermediate-onset CRC in terms of survival as well.

In summary, we described differential clinicopathological and molecular features of a group of Chilean patients with CRC according to the age of onset and found that the EOCRC group was characterized by a family history of cancer, left-sided tumors with a more advanced stage of the disease but better survival at 10 years, and lower MSI, with predominant germline mutations.

We encourage other Latin American research groups to realize further characterization of CRC, in order to better understand carcinogenic pathways that will guide new treatment approaches.

## 5. Conclusions

The increasing mortality in the EOCRC group worldwide and in Chile alerts about the necessity to do a more active assessment of CRC in the population, knowing that younger individuals are being diagnosed at later stages of the disease.

There are differential clinicopathological and molecular characteristics in CRC according to the age of onset, which confirms the findings previously reported in other populations. EOCRC category was characterized by a family history of cancer, left-sided tumors with a more advanced stage of the disease but better survival at 10 years, with predominant germline mutations. IOCRC has shown clinical similarities with the EOCRC and molecular similarities to the LOCRC, while this latter group was characterized by right-sided tumors, with higher frequency of MSI tumors and a worse OS and CSS. The importance of this study lies in the identification of differential characteristics of CRC patients according to age of onset—this stratification will be meaningful to improve and individualize therapeutic strategies for each patient with a real impact on the CRC burden.

To our knowledge, this is the first study that analyzes multiple characteristics of Chilean patients with CRC according to age and we encourage ongoing efforts to promote universal access to molecular analysis, whose study will have an effect on the overall prevention, early diagnosis, and tailored treatment of CRC.

## Figures and Tables

**Table 1:** Clinical, pathological, and familial features of the patients.

	Number of Cases (%)			
	EOCRC	IOCRC	LOCRC	*p*-Value ^1^
Number of patients	72 (17%)	214 (50%)	140 (33%)	-
Mean age at onset in years (range)	43 (29–50)	61 (51–69)	79.8 (70–98)	-
Gender:				0.0031
Male	33 (45.8%)	135 (63.1%)	66 (46.8%)	
Female	39 (54.2%)	79 (36.9%)	74 (52.9%)	
Location				0.0132
Right colon	15 (20.8%)	62 (29.0%)	62 (42.9%)	
Left colon	41 (56.9%)	111 (51.9%)	53 (42.1%)	
Rectum	16 (22.2%)	41 (19.2%)	27 (15%)	
TNM stage				
in situ	2 (2.8%)	6 (2.8%)	3 (3.5%)	
I	12 (16.7%)	30 (14.0%)	22 (15.7%)	
II	12 (16.7%)	68 (31.8%)	46 (32.9%)	
III	26 (36.1%)	72 (33.6%)	52 (37.1%)	
IV	20 (27.8%)	37 (17.3%)	17 (12.1%)	0.0174 ^2^
Personal history of cancer ^3^				NS (0.5667)
No personal history	65 (90.2%)	174 (81.3%)	111 (79.3%)	
With personal history	7 (9.7%)	40 (18.7%)	30 (21.3%)	
CRC	2 (2.8%)	12 (5.6%)	8 (5.7%)	
Lynch-related	1 (1.39%)	8 (3.73%)	7 (5%)	
Other tumors	4 (5.55%)	20 (9.35%)	15 (10.7%)	
Family history of cancer				0.0256
No family history	56 (77.8%)	151(70.6%)	116 (82.9%)	
With family history	16 (22.2%)	63 (29.4%)	24 (17.0%)	
CRC	7 (9.72%)	22 (10.3%)	4 (2.86%)	
Lynch-related	4 (5.56%)	13 (6.00%)	7 (5%)	
Other tumors	5 (6.94%)	28 (13.1%)	13 (9.29%)	
BMI ^4^ (mean (SD))	24.40 (4.045)	26.35 (4.275)	22.44 (3.842)	0.00095 ^5^

^1^ Statistical comparison was performed using chi-square (χ2) test; ^2^ Statistical comparison was performed comparing Stage IV versus other stages; ^3^ Multiple tumors, synchronous and metachronous; ^4^ BMI: Body Mass Index (kg/m^2^); ^5^ Statistical comparison was performed using Dunn’s Multiple Comparison Test; *p* < 0.05; SD: standard deviation. NS: not significant.

**Table 2:** Colorectal diagnosis trends according to age over the years.

	Number of CRC Diagnosed (%)		
Periods of Study	EOCRC	IOCRC	LOCRC
2008–2011	16 (18%)	47 (52%)	27 (30%)
2012–2015	26 (17%)	74 (50%)	49 (33%)
2016–2019	30 (16%)	93 (50%)	64 (34%)

**Table 3:** Molecular features of tumors and germline genetic analysis of patients according to the age of onset.

	Number of Cases (%)			
	EOCRC	IOCRC	LOCRC	*p*-Value ^1^
Number of patients	72 (17%)	211 (50%)	138 (33%)	
MSI	10/69 (14%)	33/210 (16%)	31/137 (23%)	NS (0.1897)
MLH1 methylated	0/12 (0%)	8/69 (12%)	6/42 (14%)	NS (0.3876)
BRAF-mut (V600E)	0/28 (0%)	12/91 (13%)	9/55 (16%)	NS (0.1297)
KRAS-mut	13/33 (39%)	27/101 (27%)	21/61 (34%)	NS (0.3225)
CRC hereditary criteria				
Amsterdam	7 (9.7%)	6 (2.84)	1 (0.72%)	
FAP	3 (4.2%)	-	-	
Germline mutations	9/14 (64%)	5/18 (27.7%)	-	
APC	3	0	-	
MLH1	3	1	-	
MSH2-EPCAM	2	2	-	
PMS2	1	2	-	

^1^ Statistical comparison was performed using chi-square (χ2) test. MSI: microsatellite instability. FAP: Familial Adenomatous Polyposis. NS: not significant.

**Table 4:** Survival analysis according to the age of onset.

	OS				CSS			
	5-Years Survival %	95% CI	10-Years Survival %	95% CR	5-Years Survival %	95% CI	10-Years Survival %	95% CR
EOCRC	73.6	[61.8–87.7]	61.5	[45.2–83.7]	77.2	[65.6–90.8]	69.4	[53.4–90.3]
IOCRC	80.0	[72.9–87.8]	35.9	[14.1–91.2]	95.9	[79.9–92.4]	63.0	[39.6–100]
LOCRC	48.5	[38.3–61.5]	35.1	[23.1–53.1]	68.7	[58.8–80.1]	68.7	[58.8–80.1]

OS: overall survival. CSS: cancer-specific Survival. SD: standard deviation.
